# Longitudinal Examination of Sexual Risk Behavior in College Students With and Without Attention-Deficit/Hyperactivity Disorder

**DOI:** 10.1007/s10508-023-02660-0

**Published:** 2023-08-07

**Authors:** Lisa Weyandt, George J. DuPaul, Emily Shepard, Jeffrey D. Labban, Alyssa Francis, Avery Beatty, Arthur D. Anastopoulos

**Affiliations:** 1 Department of Psychology, Interdisciplinary Neuroscience Program, University of Rhode Island, Kingston, RI 02881 USA; 2 College of Education, Lehigh University, Bethlehem, PA USA; 3 School of Health and Human Sciences, University of North Carolina Greensboro, Greensboro, NC USA

**Keywords:** ADHD, Young adults, College students, Sexual risk, Substance use

## Abstract

The present study sought to identify differences in the rates and predictors of risky sexual behavior among college students with and without attention-deficit hyperactivity disorder (ADHD). Current ADHD diagnosis, medication status among those with ADHD, executive functioning, substance use, comorbid anxiety, comorbid depression, and gender were identified as potential predictors of increased risky sexual behavior. Multiple group latent growth curve modeling was used to estimate trajectories of risky sexual behavior across four years of college among college students with ADHD (*n*_medicated_ = 99, *n*_unmedicated_ = 105) and a comparison group (*n* = 217) recruited from colleges throughout the eastern United States (M age = 18.23 years, 53% female, 70% White). First-year college students with ADHD reported significantly higher rates of sexual risk behavior than their peers without ADHD, with no significant differences found based on medication status. Students with ADHD who were taking medication for ADHD reported significant decreases in risky sexual behavior over time. Among college students with ADHD, anxiety was related to increased current risky sexual behavior in the medicated group, while depression was predictive of decreased future risky sexual behavior in the unmedicated group. Alcohol and cannabis use were significantly associated with increased mean levels of risky sexual behavior across all three groups, and cannabis use was associated with decreased future risky sexual behavior within the comparison group. Executive functioning deficits and male gender were predictive of risky sexual behavior within the comparison group. The results demonstrate that college students with ADHD, regardless of medication status, are at an increased likelihood of engaging in risky sexual behavior.

## Introduction

Attention-deficit/hyperactivity disorder (ADHD), a neurodevelopmental disorder characterized by deficits in sustained attention, hyperactivity, and impulsivity, is estimated to affect 2–4% of the adult population (American Psychiatric Association [APA], 2013; Barkley et al., 2006). For decades, it was purported that children with ADHD would outgrow their symptoms with the onset of puberty; however, follow-up studies have found that most individuals who are diagnosed with ADHD in childhood continue to display symptoms of the disorder into adolescence and adulthood (Barkley et al., 2003; Cherkasova et al., 2022; DuPaul et al., 1991; Swanson et al., 2017). Compared to the child literature, less information is available pertaining to adults who continue to meet criteria for an ADHD diagnosis, especially among the college student population. This relative lack of information is concerning as existing research supports that college students currently diagnosed with ADHD are at risk for academic difficulties, anxiety disorders, psychological adjustment difficulties, and problems with internal restlessness (DuPaul et al., 2021; DuPaul & Weyandt, 2006; Gormley et al., 2019; Green & Rabiner, 2012; Norvilitis et al., 2008; O’Rourke et al., 2020; Rabiner et al., 2008; Weyandt et al., 2017). Preliminary studies also suggest that college students with ADHD are at greater risk for engaging in risk-taking behavior, including sexual risk behavior (Loya et al., 2019; Pollak et al., 2019; Rohde et al., 2018).

Sexual risk behavior has been defined as “any sexual behavior (e.g., unsafe sex) that increases the likelihood of negative health outcomes, including sexually transmitted infections (STIs) such as human immunodeficiency virus (HIV), as well as unwanted pregnancies” (Cooper, 2002, p. 101; Tilahun & Mamo, 2020). Follow-up studies of adults with ADHD have found that they are at greater risk for engaging in risky sexual behaviors, such as early age of sexual intercourse, infrequent use of condoms and other forms of birth control, a high number of sexual partners, and increased risk for STIs (Flory et al., 2006; Hechtman et al., 2016; Meinzer et al., 2020). A greater level of understanding regarding sexual risk behavior among young adults, particularly undergraduate college students, is imperative, as risky sexual behavior is prevalent during this developmental period (Fergus et al., 2007). For example, the American College Health Association (2018) found that among college students who were sexually active, 8.9% reported having had four or more sexual partners in the last year. Among college students who reported ever having sex, 72.2% engaged in risky sexual behaviors in the last 12 months (Yang et al., 2019). It has been documented that students engaging in risky sexual behavior are more likely to experience adverse consequences, such as STIs and/or unwanted pregnancies (CDC, 2018). Indeed, research has found that only 77% of sexually active undergraduates reported consistent condom use within the last month (American College Health Association, 2018). Further, the Centers for Disease Control and Prevention (CDC) reported that there are 10 million new STI cases each year among individuals aged 15–24 and that there has been a 19% increase in specific STIs (i.e., chlamydia and congenital syphilis) among this group between 2014 and 2018 (CDC, 2018). These findings underscore the importance of investigating predictors of sexual risk behavior in the 12.3 million college students in America who are under 25 years of age (National Center for Education Statistics, 2018).

Although studies have explored sexual risk behavior among the general college student population, a dearth of studies has investigated sexual risk behavior in college students with ADHD relative to their neurotypical peers, even though ADHD is commonly characterized by deficits in self-regulation. Indeed, preliminary studies have found that childhood ADHD predicts risky sexual behavior in adulthood (e.g., Carlander et al., 2022; Flory et al., 2006), and more recent results from the multimodal treatment study of ADHD revealed that those with childhood ADHD were at twofold increased risk of early pregnancy (Meinzer et al., 2020). Furthermore, higher rates of STIs are evident in adults with a childhood history of ADHD compared to adults with no history of ADHD (Ramos Olazagasti et al., 2013). Young adults and adolescents with ADHD are more likely to contract HIV, syphilis, genital warts, gonorrhea, chlamydial infections, and trichomoniasis than their neurotypical counterparts (Chen et al., 2018). Additionally, longitudinal research has shown that young adults with a history of ADHD were more likely than control participants to become parents at a younger age (38 vs. 4%) and to have been treated for STIs (16% vs. 4%) (Barkley et al., 2006; Carlander et al., 2022). These findings highlight that college students with ADHD may be at an increased risk for engagement in risky sexual behavior and are a critical population to study.

Predictors of risky sexual behavior (e.g., inconsistent use of contraception, multiple sexual partners) are important to consider in order to adequately understand these risk behaviors and inform appropriate and effective interventions for college students with ADHD. Numerous studies have found that substance use (e.g., alcohol, cannabis) commonly occurs before, or in combination with, sexual activity among college students (American College Health Association, 2016; Kilwein & Looby, 2018; Wang et al., 2018). For example, studies have found that risky sexual behavior is common following alcohol consumption (Kilwein & Looby, 2018; Yang et al., 2019), and Patrick et al. (2015) reported that on a given day, consuming more drinks and binge drinking is associated with greater odds of unprotected oral and penetrative sex and other risky sexual behaviors. Brown and Vanable (2007) also found that strong social motives significantly increase the odds of engaging in risky sexual behaviors. Specifically, they found that the relationship between alcohol consumption and unprotected vaginal sex was evident among individuals in a non-steady relationship, but not among those in a committed relationship. Similar results have been found between cannabis use and risky sexual behavior in adults, with frequent cannabis use predictive of unsafe sex practices, such as infrequent condom use (Buckner et al., 2018). These findings are especially relevant among college students with ADHD, as these students often report greater cannabis and alcohol use compared to their neurotypical peers (Chen et al., 2018), and consequently may be at increased risk for engagement in risky sexual behavior.

In addition to substance related issues, the extant research demonstrates that sexual risk behavior among college students is associated with mental health disorders that are frequently comorbid with ADHD (e.g., anxiety and depression) (Franke et al., 2018; O’Rourke et al., 2020). For example, Burke et al. (2018) found that female college students who reported significant levels of depression also reported engaging in a greater frequency of unprotected sex in attempts to improve their mood. Increased levels of anxiety are also associated with decreased condom use among young adult males (Hill et al., 2017). Additionally, executive functioning (i.e., the ability to plan, shift, inhibit, and regulate behaviors and impulses) is also associated with risky sexual behavior among young adults (Dir et al., 2014; Espeleta et al., 2018; Hentges et al., 2018; Regan & Tubman, 2020; Van Eck et al., 2014). Specifically, poorer executive functioning has been associated with greater sexual risk outcomes in college students (Regan & Tubman, 2020; Reynolds et al., 2019; Rosenberg et al., 2018). Impulsivity, a multifaceted construct that refers to acting in the moment without careful regard for negative consequences, has also been particularly associated with increased sexual risk behavior in young adults (Dir et al., 2014; Leeman et al., 2019; Wilson & Vassileva, 2016). Research also supports that college students with ADHD often exhibit significantly greater deficits with respect to inhibition and regulation compared to their non-ADHD peers (Hertz et al., 2022; Weyandt et al., 2017). Collectively, these findings suggest that college students with ADHD may have difficulties with inhibiting their impulses and delaying gratification, and consequently, may be more likely to engage in risky sexual behavior (Hertz et al., 2022; Weyandt et al., 2017).

Despite the growing body of research concerning young adults with ADHD, there are nonetheless significant gaps in the literature concerning longitudinal sexual risk behaviors in college students with ADHD relative to their neurotypical peers, namely whether risky sexual behaviors increase or decrease in either group during the college years (Francis et al., 2022; Nelson et al., 2019). Importantly, information is also lacking regarding variables such as medication status, executive functioning, substance use, psychiatric comorbidity (e.g., depression, anxiety), and whether gender may predict sexual risk behavior in college students with and without ADHD. Given that prescription stimulant medication may serve as a protective factor for young adults with ADHD (i.e., with respect to substance use and physical injury) (Barkley et al., 2003; Schermann et al., 2018), it is also plausible that such treatment may be associated with decreased risk of sexual risk behavior in college students with ADHD. For example, research has demonstrated that impulsivity is associated with higher sexual risk (Leeman et al., 2019; Wilson & Vassileva, 2016), and prescription stimulant medication (e.g., methylphenidate and lisdexamfetamine) contributes to decreased impulsivity symptoms (DuPaul et al., 2012; Mechler et al., 2022). Grant et al. (2018) found that misuse of prescription stimulants was associated with higher risk sexual practices (earlier age at first sexual experience and lower use of barrier contraception) among a sample of 9449 college students; however, questions remain regarding the relationship between prescription stimulant medication and sexual risk behavior among college students with and without ADHD.

The purpose of the present study was to address several gaps in the literature pertaining to college students with and without ADHD, by exploring the following research questions: How do first-year college students with and without ADHD compare with respect to sexual risk behavior(s) across 4 years of college attendance? Among medication status, executive functioning, substance use, psychiatric comorbidity, and demographic characteristics, which of these factors is associated with risky sexual behaviors over time? Given the limited longitudinal data concerning sexual risk behavior in college students with ADHD, trajectory means and associations with covariates were modeled in the absence of directional hypotheses. However, hypotheses concerning overall group mean (i.e., intercepts) differences and associations with covariates were reasonable given the previous literature. Specifically, it was hypothesized that across the 4 years of the study: (1) College students with ADHD, regardless of ADHD medication status, would report significantly higher rates of sexual risk behavior than college students without the disorder; (2) college students with ADHD who were prescribed medication would report significantly lower rates of sexual risk behavior than college students with ADHD not taking medication; (3) higher rates of executive functioning deficits, substance use, and symptoms of anxiety and depression would be associated with higher rates of sexual risk behavior for all groups; and (4) males would report significantly higher rates of sexual risk behavior than females, regardless of ADHD diagnosis or medication status.

## Method

### Participants

Participants included first-year college students who were initially screened to determine their eligibility for the Trajectories Related to ADHD in College (TRAC) project and were then followed for the duration of a 4-year multi-site longitudinal investigation. The original sample consisted of 572 first-year students; however, after screening, 456 met the study’s eligibility requirements. Included in this total were 228 students with ADHD and 228 non-ADHD comparison group students. Of the 456 participants who met the study's eligibility requirements, 35 withdrew from the study before completing the year 1 assessment process. An additional 10 participants with ADHD did not provide their initial medication status. These 10 participants were similar to the analytic sample in terms of racial and ethnic identity; however, whereas the analytic sample was approximately balanced by gender, the excluded participants were primarily male. This withdrawal of participants resulted in a final sample of 421, including 204 students with ADHD and 217 non-ADHD comparison participants, who were assessed annually across 4 years. To consider the potential impact of medications, the ADHD group was subdivided based on their medication status in assessment year 1. Medication status was defined as individuals taking stimulant or non-stimulant medication to treat symptoms of ADHD; henceforth, “medication” will indicate use of medication, stimulant or non-stimulant, for the treatment of ADHD. This division resulted in 105 ADHD participants taking medication and 99 not taking medication. Medication status was determined at Year 1 to inform interventions targeting risk factors in Year 1 that may impact trajectories throughout college. As shown in Table 1, the sample was composed of approximately 47.9% males in the non-ADHD group and 46% males in the ADHD group. Further, non-Hispanic Whites represented 63.5% and 61% of the participants in the non-ADHD and ADHD-diagnosed no medication groups, respectively, and 81.8% in the ADHD-diagnosed taking medication group. Participant characteristics were relatively consistent with the demographics of the nine universities from which they were drawn.

**Table 1:** Demographic information

	*N* = 217	*N* = 105	*N* = 99
	Comparison	ADHD Not Medicated	ADHD Medicated
Age	18.20 (0.46)	18.28 (0.53)	18.22 (0.55)
Gender (% Female)	52.10%	54.30%	54.50%
Male	104	48	45
Female	113	57	54
Race (% Caucasian)	65.90%	65.70%	86.90%
Caucasian	143	69	86
African American	30	20	5
Asian	19	3	3
More than one	7	6	1
Other/Not Reported	18	7	4
Hispanic (Total)	23	13	9
Caucasian	5	5	5
African American	0	1	0
Asian	0	0	0
More than one	2	1	0
Other/Not reported	16	6	4
IQ	110.56 (11.83)	109.10 (11.55)	112.56 (13.47)

To be eligible for the ADHD group, participants were required to meet the *Diagnostic and Statistical Manual of Mental Disorders* (DSM-5) criteria (American Psychiatric Association, 2013). ADHD status, as well as non-ADHD comparison group status, was determined based on a multi-method assessment procedure that included expert panel review. At the first stage of this assessment process, all participants initially completed a self-reported ADHD Rating Scale (DuPaul et al., 1998), that was modified to address current and past ADHD symptoms, as well as medication status. If a participant’s self-report or parent report indicated that they frequently displayed 4 or more symptoms of either inattention or hyperactivity–impulsivity during both childhood, as well as the past 6 months, a Semi-Structured Interview for Adult ADHD was administered to address full DSM-5 criteria for ADHD (i.e., 5 or more symptoms of either inattention or hyperactivity–impulsivity being present). This same process was followed for potential comparison participants (i.e., self-reported and parent-reported responses to the ADHD Rating Scale indicated the presence of 3 or fewer symptoms for both inattention and hyperactivity–impulsivity during childhood and the past 6 months). Subsequent participant interview responses that indicated the presence of 3 or fewer symptoms from both symptom lists were used to determine eligibility for the comparison group. All potentially eligible cases were then independently reviewed by a panel of four ADHD experts (i.e., the three principal investigators and a nationally recognized adult ADHD consultant). Unanimous panel agreement was required for the final determination of ADHD and comparison group status, as well as for psychiatric comorbidity status. As expected, the two groups (e.g., ADHD, comparison) differed in terms of their self-reported ADHD symptoms. Within the ADHD group, there were 48.2% with a Combined presentation, 46.8% with a predominantly inattentive presentation, and 5.0% with a predominantly hyperactive–impulsive presentation.

### Measures

#### Background Information

All participants completed a form regarding demographic and contact information. Participants also underwent a background interview to provide information about their K-12 school history, demographics, and personal and family histories of mental health difficulties. Mean scores by group for the predictor variables can be found in Table 2.

**Table 2:** Scores by group

	Comparison	ADHD Not medicated	ADHD medicated
	*N* = 217	*N* = 105	*N* = 99
SRS Total Yr1^a^	12.38 (12.84)	16.29 (13.94)	18.39 (16.70)
SRS Total Yr2	11.75 (10.85)	14.98 (10.30)	16.09 (14.19)
SRS Total Yr3	12.25 (10.67)	16.19 (13.44)	17.58 (14.28)
SRS Total Yr4	12.70 (11.36)	16.88 (13.19)	14.67 (13.46)
BDI Avg^b^	6.098 (5.00)	15.60 (8.40)	13.45 (8.53)
BAI Avg^c^	4.91 (5.56)	13.59 (9.63)	13.12 (9.79)
BRIEF Avg^d^	90.12 (14.67)	136.14 (22.52)	133.92 (20.92)
ASSIST: Alcohol Avg^e^	5.63 (4.91)	5.80 (4.23)	7.57 (6.28)
ASSIST: Cannabis Avg^e^	2.59 (4.28)	4.61 (6.72)	5.41 (6.59)

^a^Absolute range for SRS: 0–92

^b^Absolute range for BDI: 0–63

^c^Absolute range for BAI: 0–63

^d^Absolute range for BRIEF: 0–150

^e^Absolute range for ASSIST Alcohol: 0–8

^e^Absolute range for ASSIST Cannabis: 0–8

#### ADHD Rating Scale, Self-Report Version (ADHD RS-SRV)

The ADHD RS-SRV is a modified version of the ADHD RS-IV (DuPaul et al., 1998). Inattention (IN) and hyperactive–impulsive (HI) symptoms are listed in an alternating fashion, and the frequency of occurrence for each symptom is rated as: 0 (*never or rarely present*), 1 (*sometimes present*), 2 (*often present*), or 3 (*very often present*). The total number of items scored 2 or 3 yields symptom frequency counts for both IN and HI. In contrast to the original ADHD RS-IV, the ADHD RS-SRV simultaneously assesses ADHD symptoms both during childhood and during the past 6 months, while also considering medication status. Coefficient alphas based on the current sample were very good (0.74) to excellent (0.94) for the childhood and past 6 months reports of both IN and HI symptoms, regardless of medication status.

#### ADHD Rating Scale, Parent Report Version

The ADHD RS-PRV required parents to rate their child’s ADHD symptoms during both childhood and the past 6 months. For participants with histories of taking medication, parents provided ratings based on their child’s status when not taking medication. The format and scoring of the ADHD RS-PRV are like that of the ADHD RS-SRV. The ADHD RS-PRV possesses high internal consistency (0.89 to 0.94) based on the current sample.

#### Semi-Structured Interview for Adult ADHD

The Semi-Structured Interview for Adult ADHD was developed specifically for this study to assess (1) functional impairment for each of the 18 symptoms and (2) symptom frequency counts as a function of medication status. For symptoms endorsed as present “most of the time,” additional questioning examines that symptom’s impact on daily functioning. The interview also addressed the duration, age of onset, and other DSM-5 criteria for ADHD. Coefficient alphas based on the current sample were good for the IN (0.90) and HI (0.85) portions of the interview.

#### Structured Clinical Interview for DSM Disorders (SCID-I)

The SCID-I (First et al., 1996) is a psychometrically sound interview that is widely used in clinical research. The SCID-I Mood and Anxiety Disorders modules were routinely administered to all participants. Other SCID-I modules were given as needed for participants reporting a personal or family history of psychiatric disorders during the background interview.

#### Sexual Risk Survey (SRS)

The SRS (Turchick & Garske, 2008) is a 23-item questionnaire that prompted respondents to report the frequency of their participation in each of a range of sexual risk behaviors during the preceding 6 months. The SRS has excellent internal consistency (0.90) and a stable, five-factor structure (i.e., Sexual Risk Taking with Uncommitted Partners, Risky Sex Acts, Impulsive Sexual Behaviors, Intent to Engage in Risky Sexual Behaviors, and Risky Anal Sex Acts). The SRS also has high test–retest reliability (0.93) and has been found to reliably predict a global score of sexual risk (Francis et al., 2022; Turchick & Garske, 2008). To assess general risk, the total SRS score was used as the dependent measure in the present study. The mean score on the SRS within the present sample was 14.77 with a standard deviation of 14.00.

#### Behavior Rating Inventory of Executive Function-Adult Version (BRIEF-A)

Aspects of executive functioning (EF) were assessed using the BRIEF-A (Gioia et al., 2000), which is a self-report instrument that takes approximately 10 min to complete and has adequate psychometric properties. Children and adults with ADHD have been found to perform more poorly on EF measures including the BRIEF-A, relative to control participants (Nigg et al., 2006; Toplak et al., 2007). To complete the BRIEF-A, participants rate the frequency of 75 problematic behaviors over the past month on a 3-point scale (1 = *never*; 2 = *sometimes*; 3 = *often*). Higher scores indicate greater degrees of executive dysfunction. In addition to providing nine specific EF subscales, the BRIEF-A generates three general composite scores—Behavior Regulation Index (BRI), Metacognition Index (MCI), and General Executive Composite (GEC). The scores from all nine subscales form the GEC, which was used as the dependent variable in the present study. The BRIEF-A has demonstrated reliability, validity, and clinical utility as an ecologically sensitive measure of EF in healthy respondents, as well as individuals with a range of psychiatric and neurological conditions (Gioia et al., 2000; Roth et al., 2005). Scores on the BRIEF-A within the present sample ranged from 34–96, with a mean score of 53.21 (SD = 14.32).

#### Alcohol Smoking and Substance Involvement Screening Test V3.0 (ASSIST)

Substance use (e.g., alcohol and illicit drug use) was operationalized using the ASSIST (World Health Organization [WHO], 2002), a validated screening instrument for determining an individual’s substance use patterns (Humeniuk et al., 2018). The ASSIST covers 10 substances: alcohol, cannabis, cocaine, amphetamine-type stimulants, inhalants, sedatives, hallucinogens, opioids, and “other drugs.” “Other” drugs are those that do not fit in with other psychoactive substances (World Health Organization [WHO], 2002). For example, khat, nutmeg, and caffeine can be categorized under “other.” The questions from ASSIST assess lifetime and current use of substances and individuals respond using Likert scale options assessing quantity and frequency of use, as well as the degree to which use is problematic. The ASSIST has a sensitivity ranging from 54 to 94% and specificity ranging from 50 to 96%. The assessment also has a good to excellent test–retest reliability ranging from 0.58 to 0.90 depending on the substance, and construct validity ranging from 0.77 to 0.94 (Humeniuk et al., 2018). The use of alcohol and cannabis were analyzed separately. Due to the low incidence of use of the other substances in this sample, all the other substances were analyzed as one “other” category. Scores on the ASSIST as it relates to alcohol use ranged from 0 to 29 with a mean score of 5.89 (SD = 6.13). Regarding cannabis use, scores ranged from 0 to 38 with a mean score of 3.63 (SD = 6.28).

#### Beck Depression Inventory-II (BDI-II)

The BDI-II is a self-report measure of depression that is psychometrically sound and has been widely used in research and clinical practice (Beck et al., 1996). The total score from the BDI-II was used as a dimensional measure of depression. The mean score of the BDI-II within the current sample was 10.70 (SD = 9.08).

#### Beck Anxiety Inventory (BAI)

The BAI is often used in research and clinical practice and possesses adequate psychometric properties (Beck & Steer, 1993). The total score from the BAI was used as a dimensional measure of anxiety. The mean score of the BAI was 9.53 (SD = 9.76).

### Procedure

The goal of the TRAC project was to examine multiple functional trajectories (e.g., educational, behavioral, social, emotional, and vocational) across this early period of emerging adulthood and to identify risk and protective factors that inform clinical assessment and treatment. Three primary sites were involved, including one university in the Southeast and two universities in the Northeast United States. In addition, six colleges and universities near the primary sites served exclusively as recruitment sites. To achieve recruitment goals, two cohorts of first-year students were recruited successively across the first 2 years of the project and were assessed annually until graduation. A total of 219 participants were recruited in Cohort 1 and another 237 participants were recruited in Cohort 2. All participants underwent an annual four-stage assessment process, for which they earned up to $100 as an incentive for completing all required procedures. Recruitment and data collection occurred continuously throughout the fall and spring semesters at times that were convenient to each participant. Participants were recruited from multiple sources, including summer orientation sessions, the Disability, Access, and Inclusion office, student counseling centers, fliers, and presentations to large first-year classes.

First-year college students who were between 18 and 25 years of age and entering college for the first time were recruited, provided informed consent, and underwent subsequent annual assessments for 4 years. The data collected by the self-report and parent report of the ADHD RS served as the basis of decisions regarding which participants were excluded from the study and which moved to the next phase of assessment. Participants whose ADHD RS score indicated possible inclusion underwent additional evaluation by the Semi-Structured Interview for Adult ADHD and the Structured Clinical Interview for DSM-5 (SCID-5), which informed decisions pertaining to which cases were brought to the expert panel for review and final determination of ADHD or comparison group classification, as well as psychiatric comorbidity status. To be eligible for the study, participants either met a full DSM-5 diagnosis for ADHD by demonstrating five or more symptoms of inattention or hyperactivity, or they met criteria for the comparison group by demonstrating three or fewer symptoms of inattention or hyperactivity characterized by ADHD, both during childhood and in the past 6 months. The expert panel was comprised of four doctoral-level, licensed psychologists with expertise and experience in ADHD. Group assignment required unanimous agreement by the panel.

All data were collected by predoctoral- and doctoral-level staff from clinical psychology and school psychology backgrounds. All staff received extensive training before the start of the project, and their adherence to the various assessment protocols was monitored on an ongoing basis to maintain consistency across sites. Study procedures were reviewed on an annual basis and were approved by the Institutional Review Boards at each site.

All statistical modeling was conducted using Mplus software, version 8 (Muthén & Muthén, 1998–2017). Multiple group latent growth curve modeling (LGCM) was used to estimate trajectories of change in self-reported risky sexual behavior as measured by the SRS. In this model, covariates are treated as predictors as is an established approach in the literature (DuPaul et al., 2021; Muniz-Terrera et al., 2017; Kaczmarek & Trambacs-Oleszak, 2017). Given the consistently spaced annual assessment schedule, the time metric was set at 1-year intervals and the intercept was set at year 1.

#### Data Analytic Approach

Visual inspection of the group means over time suggested trajectories were linear as opposed to nonlinear. Therefore, we proceeded to fit a linear conditional growth model to the data. The presence of three groups—non-ADHD comparisons, ADHD-diagnosed taking ADHD medication, and ADHD-diagnosed not taking ADHD medication—allowed us to take advantage of a multiple group approach (Muthén & Muthén, 1998–2017), in which separate latent curve growth models are simultaneously fit to each subgroup. This approach allowed for the estimation of linear growth and testing of model constraints unique to each subgroup, while also yielding fit statistics for the omnibus model. The model was fit using full information maximum likelihood estimation, which allowed for the inclusion of all data, including cases with incomplete data. Although psychological and behavioral covariates were assessed each year, it was determined that cross-sectional values at each time point might be overly state-dependent. Therefore, each participant’s scores for measures of executive function, anxiety, depression, and alcohol and cannabis use were averaged across their available observations and entered into the model as time-invariant covariates. However, preliminary analyses in which psychological and behavioral variables were treated as time-varying covariates were conducted to ensure there were no substantive differences in results. In addition to the aforementioned independent variables, participant gender and race/ethnicity were also included in the model (i.e., regressed onto intercept and slope). All covariates were grand-mean-centered, such that scores used in the analyses represented individual’s deviations from the sample means. We then took steps to refine the conditional model. Growth factors yielding small, nonsignificant estimates were fixed to 0, and likelihood ratio tests were conducted to ensure fit was not significantly reduced. Once the model was finalized, equality constraints were used to test for between-groups differences in growth factor means and variances.

Omnibus model fit was assessed via examination of multiple criteria: nonsignificant chi-square statistic, RMSEA < 0.08, CFI > 0.95, and SRMR ≤ 0.08. Due to the non-normal nature of the outcome data, we used bootstrapping (*N* = 10,000) to calculate bias-corrected confidence intervals when making determinations of statistical significance: 95% CI (CI_95_) for growth parameters and 90% CI (CI_90_) for covariates. Therefore, statements regarding statistical significance refer to instances in which the confidence intervals did not include 0. Alternatively, between-groups comparisons of growth factors were made using the Wald Chi-square test of parameter equalities (Asparouhov & Muthén, 2010), and thus, *p*-values are reported along with statements regarding the significance of these results. For between-groups comparisons at specific years, the final model was refit with time-centered such that intercepts would be estimated at the year of interest and equality constraints were retested.

## Results

### Sexual Risk Survey Model

Fit statistics for the omnibus model suggested adequate-to-good fit:* χ*^2^(68) = 93.23, *p* = 0.023; RMSEA = 0.051 (CI_90_ = 0.020, 0.076); CFI = 0.970; SRMR = 0.059. At the group level, model fit for the non-medicated ADHD group was mediocre (χ^2^(23) = 37.81, *p* = 0.02; RMSEA = 0.078), while fit was good for both the medicated ADHD group ( χ^2^(22) = 22.60, *p* = 0.42; RMSEA = 0.017) and the non-ADHD group (*χ*^2^(23) = 32.82, *p* = 0.08; RMSEA = 0.044). Slope estimates for both the comparison and non-medicated ADHD groups were small and nonsignificant; therefore, these growth terms were fixed to 0 and did not diminish model fit (LRT *p* = 0.574). See Table 3 for full model results.

**Table 3:** Full latent growth curve modeling results

		Non-ADHD			ADHD medicated			ADHD not medicated	
	Estimate	SE	CI_95_	Estimate	SE	CI_95_	Estimate	SE	CI_95_
Intercept	12.38*	0.47	11.43, 13.37	18.03*	1.25	15.55, 20.61	16.00*	0.83	14.41, 17.83
Gender	1.675	1.28	−0.41, 3.77	1.64	2.81	−3.31, 6.70	1.12	2.14	−2.46, 5.12
Race/ethnicity	1.04	1.29	−1.07, 3.06	−0.36	3.30	−6.87, 5.62	−0.93	2.17	−4.55, 2.69
EF	−0.10*	0.05	−0.19, -0.00	−0.04	0.08	−0.18, 0.12	0.06	0.06	−0.03, 0.17
Anxiety	0.14	0.17	−0.13, 0.38	0.34*	0.19	0.05, 0.68	−0.09	0.16	−0.43, 0.31
Depression	0.10	0.17	−0.19, 0.39	−0.24	0.21	−0.67, 0.15	0.05	0.20	−0.31, 0.49
Alcohol	0.70*	0.15	0.45, 1.00	1.00*	0.25	0.58, 1.50	1.35*	0.27	0.82, 1.90
Cannabis	1.07*	0.17	0.71, 1.42	0.74*	0.230	0.27, 1.21	0.51*	0.18	0.07, 0.93
Res. var	43.10*	7.71	30.22, 63.40	100.91*	22.74	63.91, 168.10	65.51*	15.90	39.93, 124.90
Slope	0	0	–	−1.19*	0.48	−2.14, -0.23	0	0	–
Gender	1.14*	0.58	0.13, 2.13	0.13	1.07	−1.68, 2.09	0.92	0.98	−0.77, 2.46
Race/ethnicity	0.13	0.58	−0.80, 1.08	−1.91	1.32	−4.23, 0.16	−0.84	0.97	−2.67, 0.94
EF	0.01	0.03	−0.04, 0.05	0.01	0.04	−0.06, 0.08	0.03	0.03	−0.01, 0.07
Anxiety	0.03	0.08	−0.10, 0.15	−0.06	0.07	−0.18, 0.05	0.19	0.08	−0.01, 0.40
Depression	0.04	0.08	−0.09, 0.17	0.05	0.08	−0.08, 0.19	−0.22*	0.10	−0.43, -0.03
Alcohol	0.043	0.07	−0.10, 0.18	−0.06	0.101	−0.25, 0.11	−0.03	0.12	−0.29, 0.169
Cannabis	−0.29*	0.08	−0.45, -0.13	−0.13	0.09	−0.28, 0.02	−0.10	0.08	−0.259, 0.08
Res. var	5.57*	1.64	2.56, 9.95	4.68	3.47	−2.18, 14.67	6.84*	3.25	1.69, 16.52

**Fig. 1:**
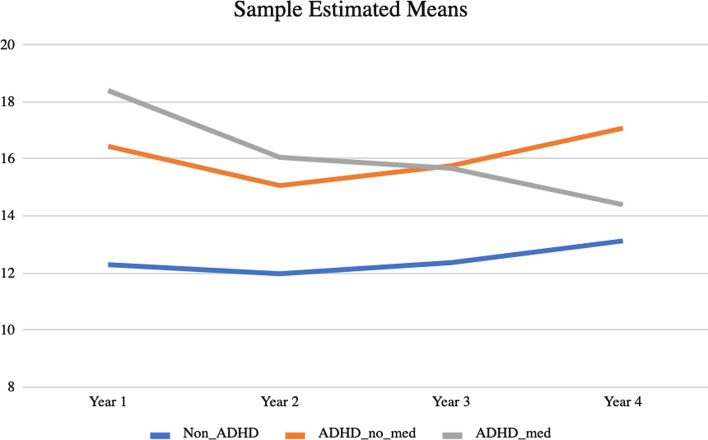
Observed change in risky sexual behavior over time

The results of the present study support the hypothesis that college students with ADHD would report significantly higher rates of sexual risk behavior than college students without ADHD (see Fig. 1). Specifically, comparisons using the Wald chi-square test indicated that both the medicated and non-medicated ADHD groups reported significantly higher rates of sexual risk behavior at each year compared to the non-ADHD comparison group (*χ*^2^(1) > 14.0, *p* < 0.001).

The hypothesis that college students with ADHD who were prescribed ADHD medication would report significantly lower rates of sexual risk behavior than college students with ADHD not taking medication was not supported. Although differences in estimated mean rates of risky sexual behavior between the ADHD groups failed to achieve significance for any year (*p* > 0.17), there was a significant decrease in risky sexual behavior over time observed in the group with ADHD taking medication (*β* =  − 1.191, CI_95_: − 2.135, − 0.227). This decline, however, did not result in sufficient change for year 4 rates to deviate significantly from the rates reported by the unmedicated group with ADHD. Slope estimates for the comparison group and the ADHD group not taking medication were trivial, such that fixing them to zero did not significantly alter model fit (χ^2^ (*df* = 2) = 1.11, *p* = 0.574).

The findings revealed that executive functioning was negatively associated with sexual risk behavior (i.e., poorer EF was associated with higher levels of sexual risk behavior) only in the comparison group (*β* =  − 0.097, CI_90_: − 0.185, − 0.003). In contrast, both alcohol and cannabis use were significantly associated with increased mean levels of sexual risk behavior at intercept (Year 1) for all three groups (see Table 3 for estimates and CI_90_). Notably, cannabis use was negatively associated with change in sexual risk-taking over time for the comparison group (*β* =  − 0.294, CI_90_: − 0.445, − 0.131) and approached significance for medicated ADHD participants (*β* =  − 0.129, CI_90_: − 0.275, 0.017). Anxiety and depression symptoms were not consistently associated with either mean levels of sexual risk behavior or change over time. Anxiety symptoms were only significantly associated with the intercept mean for the ADHD group taking medication (*β* = 0.339, CI_90_: 0.046, 0.677), and depression symptoms were only significantly associated with the slope mean for the ADHD group not taking medication (*β* =  − 0.215, CI_90_: − 0.432, − 0.029). Anxiety was associated with increased mean sexual risk behavior in the medicated ADHD group, while depression was associated with decreased mean sexual risk behavior in the unmedicated group.

Regarding potential gender differences, binary gender was not found to be consistently related to either mean rates of sexual risk behavior or change in rates of risky sexual behavior over time. Only in the comparison group did male students report greater increases in rates of sexual risk behavior over time compared to their female counterparts (*β* = 1.136, CI_90_: 0.125, 2.125).

## Discussion

The purpose of the present study was to explore the developmental trajectory of sexual risk behavior in college students with and without ADHD over 4 years, beginning in their first year of college. Specifically, the study sought to identify factors that may either predict or potentially protect against sexual risk behavior among college students with and without ADHD, including ADHD medication status, executive functioning, substance use, and comorbid psychological functioning. The first hypothesis that college students with ADHD would report significantly higher rates of sexual risk behavior than college students without the disorder across all 4 years was supported. These findings are consistent with results from cross-sectional studies, such as Huggins et al. (2015) who found that female college students with ADHD were more likely to engage in unprotected sex than their counterparts without the disorder. Flory et al. (2006) also found that individuals who had been diagnosed with ADHD were more likely than those without ADHD to engage in risky sexual behaviors, including having more sexual partners, engaging in casual sex more frequently, having an STI, or using birth control methods inconsistently. Interestingly, Berry et al. (2021) recently found that individuals with ADHD were more likely to discount the use of condoms if there would be a delay in acquiring the condoms, suggesting that students with ADHD may have difficulties with inhibiting their impulses, and consequently, delaying gratification. What is unique about the present study is that the results indicate college students with ADHD engage in significantly higher rates of risky sexual behavior beginning in the first year of college, and these behaviors continue across the 4 years. These findings highlight the need for additional research and targeted interventions to help reduce sexual risk behavior among college students, particularly among those with ADHD.

### Changes in Sexual Risk Behavior

Regarding sexual risk behavior over time, rates of sexual risk behavior remained relatively stable for the comparison and non-medicated ADHD subgroup; however, medicated students with ADHD reported a significant decline in sexual risk behavior across the 4 years. Importantly, despite the overall decrease in sexual risk behavior seen in medicated students with ADHD, the difference in overall levels of risky sexual behavior was not statistically significant between the two groups of students with ADHD. Even within the medicated group, rates of risky sexual behavior were still significantly greater than college students without ADHD. Thus, these findings only partially supported the hypothesis that college students with ADHD who took ADHD medication would report significantly lower rates of sexual risk behavior over time compared to college students with ADHD who do not take medication.

The present findings appear to suggest that ADHD medication may have a protective effect on the behavioral functioning of college students with ADHD over time, similar to studies that found an association between stimulant medication, specifically, and improved behavioral functioning in addition to neuropsychiatric functioning (Chang et al., 2019). However, future studies must explore whether prescription stimulant or non-stimulant medication affects sexual risk behavior, as well as identify possible underlying mechanisms. For example, previous research has found a negative relationship between ADHD symptomatology and decision making (e.g., Lichtenstein et al., 2012; Schepman et al., 2012) and an improvement in decision making with prescription stimulants (e.g., Mowinckel et al., 2015).

### Substance Use

The findings of the current study are consistent with the extant literature concerning alcohol and cannabis use, as well as sexual risk behavior among college students. Specifically, alcohol use was significantly associated with greater mean levels of risky behavior at intercept (Year 1) across all participant groups (comparison *β* = 0.703, CI_90_: = 0.449, 1.001; medicated ADHD *β* = 0.997, CI_90_: = 0.577, 1.495; non-medicated ADHD groups *β* = 1.349, CI_90_: = 0.822, 1.902). The relationship between cannabis and risky sexual behavior, however, was somewhat more complex. Across all three groups, cannabis use was associated with heightened mean risky sexual behavior at intercept; however, for the comparison group, cannabis use was predictive of decreased further risky sexual behavior (*β* =  − 0.294, CI_90_: − 0.445, − 0.131), running contrary to the expectations of the current study. This pattern was not seen in either ADHD group. Previous research has demonstrated that including alcohol and cannabis-related topics in interventions directed at reducing risky sexual behavior in young adults was more effective than those that did not include these topics (Bryan et al., 2018). The results of the present study are therefore pertinent to collegiate interventions, as they indicate that alcohol use, but not independent cannabis use, is predictive of risky sexual behavior. This finding is in line with Metrik et al. (2012), who found that active THC is associated with decreased impulsivity on laboratory measures of executive functioning. Among individuals with ADHD, however, cannabis use was predictive of increased risky sexual behavior at baseline without a significant decrease over time. Interventions aimed at reducing risky sexual behavior specifically among college students with ADHD may therefore benefit from targeting cannabis use.

### Psychological Functioning

In terms of psychological functioning, higher anxiety levels were associated with greater current sexual risk behavior for the medicated ADHD group (*β* = 0.339, CI_90_: 0.046, 0.677), whereas depression symptoms were predictive of lower rates of future risky sexual behavior for the non-medicated ADHD group (*β* =  − 0.215, CI_90_: − 0.432, − 0.029). Multiple mechanisms have been proposed to explain the influence of anxiety and depression on sexual risk behavior, including impaired decision making, poor coping skills, and substance use (Agardh et al., 2012; Bachanas et al., 2002; Hill et al., 2017; Turner et al., 2011). For example, Kim and Miller (2020) recently found that insecure, anxious attachment styles were significantly associated with risky sexual behavior. The present study also found a negative relationship between depressive symptoms and sexual risk behavior and can be interpreted as being in line with the previous literature supporting a relationship between depression, loss of libido, and decreased interest in sexual activity (Otte et al., 2016; Risal et al., 2016). Alternatively, other studies with general adult population participants have found both anxiety and depressive symptoms are associated with risky sexual behavior (Coyle et al., 2019).

Regarding neuropsychological functioning, executive functioning was only significantly related to risky sexual behavior in the comparison group (*β* =  − 0.097, CI_90_: − 0.185, − 0.003), and served as a significant protective factor, with increased executive functioning ability associated with decreased sexual risk. The ADHD groups, contrary to our hypotheses, did not show a significant association between executive functioning ability and risky sexual behavior. These findings were unexpected given previous research that supported the effectiveness of prescription stimulants (e.g., lisdexamphetamine) in improving executive functioning (DuPaul et al., 2011). Although speculative, it is possible that students with ADHD in the current study underestimated their executive functioning deficits, or that participants in previously published studies had greater executive functioning deficits than was self-reported. Additionally, the current study may be underpowered to detect the influence of executive dysfunction beyond that which is expected of individuals with ADHD.

### Demographic Factors

Regarding demographic factors, as hypothesized, males in the comparison group reported significant increases in risky sexual behavior over time (*β* = 1.136, CI_90_: 0.125, 2.125), but there was no significant difference across binary gender in the other groups. These results are comparable to those found recently in similar groups of college students (Gräf et al., 2020). Further, race and ethnicity were not significantly associated with sexual risk behavior for any of the groups, which differs from prior research in which women from minoritized racial and ethnic backgrounds were more likely to report STIs, but fewer sexual partners (Norris et al., 2019). These findings underscore the importance of future studies to investigate whether some elements of risky sexual behavior are associated with race and ethnicity, while others are not.

### Strengths and Limitations

Several strengths of the present study should be highlighted. First, the design of the study was unique given its diagnostic rigor, achieved by an expert panel consisting of four licensed psychologists who are specialists in the field of ADHD. Each potential participant was reviewed by the panel and unanimous agreement was needed to be included in the study. Second, this is the first study to employ a longitudinal design to study risky sexual behavior in college students with and without ADHD across a 4-year time period, enabling the examination of developmental trajectories.

Despite the study’s strengths, several limitations are also evident. First, reliance on self-report may have been subject to feigning or other response biases. Another limitation of the study included a lack of comparison of ADHD against other mental health conditions. Additionally, the present study included all types of ADHD medication (i.e., stimulant and non-stimulant) without differentiating outcomes between forms of medication. Adherence to medication was not tracked throughout the 4 years; participants were categorized by medication status in Year 1. This approach is limited because participants may have begun or ceased medication for ADHD over the course of the 4 years. Future research should aim to identify the independent contributions of stimulant and non-stimulant medications to the mitigation of risky sexual behavior, as well as identify potentially effective doses of these medications.

Additionally, the sample was comprised of students who volunteered to participate in the study; therefore, participants may differ from the larger population of college students on several variables, such as ADHD symptomatology and sexual risk behavior, and may therefore limit the generalizability of the findings. Only binary male and female responses were collected, limiting the generalizability of results based on sex. Future studies should recruit a more diverse sample of college students within and outside of the USA to address the present restrictions in the generalizability of results. Future research should also aim to compare ADHD with other mental health conditions to further clarify whether heightened risky sexual behavior is specific to ADHD, whether ADHD is contributing to sexual risk-taking behavior in isolation, or whether the comorbidity of other mental illnesses contributes to risk-taking behaviors in college students.

### Conclusion

As more students with ADHD attend college (Antschel, 2016; DuPaul et al., 2021; Weyandt & DuPaul, 2013), college students with ADHD are an important population of study. The present study examined whether college students with ADHD were more likely than their peers without ADHD to engage in risky sexual behavior and whether individuals with ADHD who were prescribed ADHD medication engaged in less risky sexual behavior than college students who were not medicated. Results revealed that college students with ADHD, regardless of medication status, engaged in greater risky sexual behavior than their peers without ADHD. This finding was consistent upon entrance into college and across their subsequent 4 years of college. In contrast to stable rates of risky sexual behavior displayed by the comparison and non-medicated ADHD groups, the medicated ADHD group displayed a significant *decline* in risky sexual behavior across the 4 years of the study. Furthermore, alcohol use was related to increased risky sexual behavior among all groups of college students; however, higher rates of cannabis use were associated with reduced risky sexual behavior in college students with ADHD who were medicated and the comparison group.

Overall, the results of the present study revealed that college students with ADHD were engaging in risky sexual behavior to a greater degree compared to their non-ADHD counterparts, and therefore, consideration of this increased risk is warranted during students’ transition to college and throughout their college years. Additional research is warranted to assess intervention techniques for monitoring and promoting healthy and safe behavior among college students with ADHD. Although the current study did not fully support that medication enhances cognitive decision making (i.e., slopes differed but means did not), additional research in an experimental context may help to clarify this issue (e.g., Christman et al., 2004).

Additionally, the results of the present study demonstrate that medicated individuals with ADHD exhibiting greater symptoms of anxiety are more likely to engage in risky sexual behavior, while unmedicated individuals with greater symptoms of depression are less likely to engage in risky sexual behavior. These findings suggest that prescription medication may have possible protective effects; however, these relationships require additional exploration as they relate to sexual behavior. It is recommended that future research promote a greater understanding of risky behavior in college students with ADHD and, importantly, design and evaluate interventions that will improve outcomes in this population. The present finding suggests that, among college students without ADHD, cannabis use may not be an important target of interventions designed to reduce risky sexual behavior. Among individuals with ADHD, however, cannabis use was predictive of increased risky sexual behavior at baseline without a significant decrease over time. Interventions to reduce risky sexual behavior specifically among college students with ADHD may therefore benefit from targeting cannabis use. Lastly, the present findings address important gaps in the literature and indicate that college students with ADHD, regardless of medication status, engaged in greater risky sexual behavior than their peers without ADHD.

## Data Availability

Data are available upon request.
